# Use of mRNA COVID-19 Vaccine After Reports of Myocarditis Among Vaccine Recipients: Update from the Advisory Committee on Immunization Practices — United States, June 2021

**DOI:** 10.15585/mmwr.mm7027e2

**Published:** 2021-07-09

**Authors:** Julia W. Gargano, Megan Wallace, Stephen C. Hadler, Gayle Langley, John R. Su, Matthew E. Oster, Karen R. Broder, Julianne Gee, Eric Weintraub, Tom Shimabukuro, Heather M. Scobie, Danielle Moulia, Lauri E. Markowitz, Melinda Wharton, Veronica V. McNally, José R. Romero, H. Keipp Talbot, Grace M. Lee, Matthew F. Daley, Sara E. Oliver

**Affiliations:** ^1^CDC COVID-19 Response Team; ^2^Franny Strong Foundation, West Bloomfield, Michigan; ^3^Arkansas Department of Health; ^4^Vanderbilt University School of Medicine, Nashville, Tennessee; ^5^Stanford University School of Medicine, Stanford, California; ^6^Institute for Health Research, Kaiser Permanente Colorado, Denver, Colorado.

In December 2020, the Food and Drug Administration (FDA) issued Emergency Use Authorizations (EUAs) for the Pfizer-BioNTech COVID-19 (BNT162b2) vaccine and the Moderna COVID-19 (mRNA-1273) vaccine,^†^ and the Advisory Committee on Immunization Practices (ACIP) issued interim recommendations for their use in persons aged ≥16 years and ≥18 years, respectively.^§^ In May 2021, FDA expanded the EUA for the Pfizer-BioNTech COVID-19 vaccine to include adolescents aged 12–15 years; ACIP recommends that all persons aged ≥12 years receive a COVID-19 vaccine. Both Pfizer-BioNTech and Moderna vaccines are mRNA vaccines encoding the stabilized prefusion spike glycoprotein of SARS-CoV-2, the virus that causes COVID-19. Both mRNA vaccines were authorized and recommended as a 2-dose schedule, with second doses administered 21 days (Pfizer-BioNTech) or 28 days (Moderna) after the first dose. After reports of myocarditis and pericarditis in mRNA vaccine recipients,^¶^ which predominantly occurred in young males after the second dose, an ACIP meeting was rapidly convened to review reported cases of myocarditis and pericarditis and discuss the benefits and risks of mRNA COVID-19 vaccination in the United States. Myocarditis is an inflammation of the heart muscle; if it is accompanied by pericarditis, an inflammation of the thin tissue surrounding the heart (the pericardium), it is referred to as myopericarditis. Hereafter, myocarditis is used to refer to myocarditis, pericarditis, or myopericarditis. On June 23, 2021, after reviewing available evidence including that for risks of myocarditis, ACIP determined that the benefits of using mRNA COVID-19 vaccines under the FDA’s EUA clearly outweigh the risks in all populations, including adolescents and young adults. The EUA has been modified to include information on myocarditis after receipt of mRNA COVID-19 vaccines. The EUA fact sheets should be provided before vaccination; in addition, CDC has developed patient and provider education materials about the possibility of myocarditis and symptoms of concern, to ensure prompt recognition and management of myocarditis.

Since June 2020, ACIP has convened 15 public meetings to review data on COVID-19 epidemiology and use of COVID-19 vaccines. The ACIP COVID-19 Vaccines Work Group, comprising experts in infectious diseases, vaccinology, vaccine safety, public health, and ethics, has held weekly meetings since April 2020 to review COVID-19 surveillance data, evidence for vaccine efficacy and safety, and implementation considerations for COVID-19 vaccination programs. After reports of myocarditis, the work group met twice to review clinical trial and postauthorization safety data for myocarditis after receipt of mRNA COVID-19 vaccines. The work group also reviewed a benefit-risk assessment of myocarditis events after receipt of mRNA COVID-19 vaccines, considering recent epidemiology of COVID-19 and sequelae of COVID-19, including myocarditis and multisystem inflammatory syndrome in children (MIS-C).** The ACIP COVID-19 Vaccines Safety Technical (VaST) Work Group, comprising independent vaccine safety expert consultants, had also reviewed safety data on myocarditis after receipt of mRNA COVID-19 vaccines at its weekly meetings. The findings from the VaST and the ACIP COVID-19 Vaccines Work Group assessments, including a summary of the data reviewed, were presented to ACIP during its meeting on June 23, 2021.

Myocarditis typically occurs more commonly in males than in females, and incidence is highest among infants, adolescents, and young adults (*1*,*2*). The clinical presentation and severity of myocarditis vary among patients. Symptoms typically include chest pain, dyspnea, or palpitations, although other symptoms might be present, especially in younger children (*3*). Diagnostic evaluation might reveal an elevated troponin level or abnormal findings on electrocardiogram, echocardiogram, or cardiac magnetic resonance imaging (Table 1). Supportive therapy is a mainstay of treatment, with targeted cardiac medications or interventions as needed. Current guidelines from the American Heart Association and American College of Cardiology recommend exercise restriction until the heart recovers.^††^

**TABLE 1:** Case definitions of probable and confirmed myocarditis, pericarditis, and myopericarditis

Condition	Definition	
**Acute myocarditis**	**Probable case**	**Confirmed case**
	Presence of ≥1 new or worsening of the following clinical symptoms:*	Presence of ≥1 new or worsening of the following clinical symptoms:*
	• chest pain, pressure, or discomfort	• chest pain, pressure, or discomfort
	• dyspnea, shortness of breath, or pain with breathing	• dyspnea, shortness of breath, or pain with breathing
	• palpitations	• palpitations
	• syncope	• syncope
	OR, infants and children aged <12 years might instead have ≥2 of the following symptoms:	OR, infants and children aged <12 years might instead have ≥2 of the following symptoms:
	• irritability	• irritability
	• vomiting	• vomiting
	• poor feeding	• poor feeding
	• tachypnea	• tachypnea
	• lethargy	• lethargy
	AND	AND
	≥1 new finding of	≥1 new finding of
	• troponin level above upper limit of normal (any type of troponin)	• Histopathologic confirmation of myocarditis^†^
	• abnormal electrocardiogram (ECG or EKG) or rhythm monitoring findings consistent with myocarditis^§^	
	• abnormal cardiac function or wall motion abnormalities on echocardiogram	• cMRI findings consistent with myocarditis^¶^ in the presence of troponin level above upper limit of normal (any type of troponin)
	• cMRI findings consistent with myocarditis^¶^	
	AND	AND
	• No other identifiable cause of the symptoms and findings	• No other identifiable cause of the symptoms and findings
Acute pericarditis**	Presence of ≥2 new or worsening of the following clinical features:	
	• acute chest pain^††^	
	• pericardial rub on exam	
	• new ST-elevation or PR-depression on EKG	
	• new or worsening pericardial effusion on echocardiogram or MRI	
**Myopericarditis**	This term may be used for patients who meet criteria for both myocarditis and pericarditis.	

**Abbreviations:** AV = atrioventricular; cMRI = cardiac magnetic resonance imaging; ECG or EKG = electrocardiogram.

* Persons who lack the listed symptoms but who meet other criteria may be classified as subclinical myocarditis (probable or confirmed).

^†^ Using the Dallas criteria (Aretz HT, Billingham ME, Edwards WD, et al. Myocarditis. A histopathologic definition and classification. Am J Cardiovasc Pathol 1987; 1:3–14). Autopsy cases may be classified as confirmed clinical myocarditis on the basis of meeting histopathologic criteria if no other identifiable cause.

^§^ To meet the ECG or rhythm monitoring criterion, a probable case must include at least one of 1) ST-segment or T-wave abnormalities; 2) Paroxysmal or sustained atrial, supraventricular, or ventricular arrhythmias; or 3) AV nodal conduction delays or intraventricular conduction defects.

^¶^ Using either the original or the revised Lake Louise criteria. https://www.sciencedirect.com/science/article/pii/S0735109718388430?via%3Dihub

** https://academic.oup.com/eurheartj/article/36/42/2921/2293375

^††^ Typically described as pain made worse by lying down, deep inspiration, or cough, and relieved by sitting up or leaning forward, although other types of chest pain might occur.

As of June 11, 2021, approximately 296 million doses of mRNA COVID-19 vaccines had been administered in the United States, with 52 million administered to persons aged 12–29 years; of these, 30 million were first and 22 million were second doses. Within the Vaccine Adverse Event Reporting System (VAERS) (*4*), the national vaccine safety passive monitoring system, 1,226 reports of myocarditis after mRNA vaccination were received during December 29, 2020–June 11, 2021. Among persons with reported myocarditis after mRNA vaccination, the median age was 26 years (range = 12–94 years), with median symptom onset interval of 3 days after vaccination (range = 0–179). Among 1,194 reports for which patient age was known, 687 were among persons aged <30 years and 507 were among persons aged ≥30 years; of 1,212 with sex reported, 923 were male, and 289 were female.^§§^ Among 1,094 patients with number of vaccine doses received reported, 76% occurred after receipt of dose 2 of mRNA vaccine; cases were reported after both Pfizer-BioNTech and Moderna vaccines. Informed by early reports, CDC prioritized rapid review of myocarditis in persons aged <30 years reported during May 1–June 11, 2021; the 484 patient records in this subset were evaluated by physicians at CDC, and several reports were also reviewed with Clinical Immunization Safety Assessment Project investigators,^¶¶^ including cardiologists. At the time of this report, 323 of these 484 cases were determined to meet criteria in CDC’s case definitions for myocarditis, pericarditis, or myopericarditis by provider interview or medical record review (Table 1). The median age of the 323 patients meeting CDC's case definitions was 19 years (range = 12−29 years); 291 were male, and 32 were female. The median interval from vaccination to symptom onset was 2 days (range = 0−40 days); 92% of patients experienced onset of symptoms within 7 days of vaccination. Of the 323 persons meeting CDC's case definitions, 309 (96%) were hospitalized. Acute clinical courses were generally mild; among 304 hospitalized patients with known clinical outcomes, 95% had been discharged at time of review, and none had died. Treatment data in VAERS are preliminary and incomplete; however, many patients have experienced resolution of symptoms with conservative treatment, such as receipt of nonsteroidal antiinflammatory drugs. Follow-up is ongoing to identify and understand longer-term outcomes after myocarditis occurring after COVID-19 vaccination.

Using myocarditis cases reported to VAERS with onset within 7 days after dose 2 of an mRNA vaccine, crude reporting rates (i.e., using confirmed and unconfirmed cases) per million second dose recipients were calculated using national COVID-19 vaccine administration data as of June 11, 2021. Myocarditis reporting rates were 40.6 cases per million second doses of mRNA COVID-19 vaccines administered to males aged 12−29 years and 2.4 per million second doses administered to males aged ≥30 years; reporting rates among females in these age groups were 4.2 and 1.0 per million second doses, respectively.*** The highest reporting rates were among males aged 12−17 years and those aged 18−24 years (62.8 and 50.5 reported myocarditis cases per million second doses of mRNA COVID-19 vaccine administered, respectively). Myocarditis rates from Vaccine Safety Datalink (VSD), based on electronic health records, were also evaluated. Although numbers were too small to show rates in all subgroups by age, VSD data indicated increased risk of myocarditis in the 7 days after receipt of dose 1 or dose 2 of an mRNA COVID-19 vaccine compared with the risk 22–42 days after the second dose, particularly among younger males after dose 2 (*5*).

To assess the benefit-risk balance of mRNA vaccines in adolescents and young adults, ACIP reviewed an individual-level assessment that compared the benefits (i.e., COVID-19 infections and severe disease prevented) to the risks (number of cases of myocarditis) of vaccination, using methods similar to those described previously.^†††^ Specifically, the benefits per million second doses administered (i.e., the benefits of being fully vaccinated in accordance with the FDA EUA) were assessed, including 1) COVID-19 cases prevented based on rates the week of May 29, 2021^§§§^; 2) COVID-19 hospitalizations prevented based on rates the week of May 22, 2021^¶¶¶^; and 3) COVID-19 intensive care unit (ICU) admissions and deaths prevented based on the proportion of hospitalized patients who were admitted to the ICU or died.**** The risks were assessed as the number of myocarditis patients reported to VAERS that occurred within 7 days of receipt of a second dose of an mRNA COVID-19 vaccine per million second doses administered through the week of June 11, 2021.^††††^ The benefit-risk assessment was stratified by age group and sex. The analysis assumed 95% vaccine effectiveness^§§§§^ of 2 doses of a mRNA COVID-19 vaccine in preventing COVID-19 cases and hospitalization and assessed outcomes for a 120-day period. The 120-day period was selected because 1) no alternative vaccine options currently exist for persons aged <18 years or are expected to be available during this period, and 2) inputs regarding community transmission have high uncertainty beyond this period, particularly in the context of circulating variants.^¶¶¶¶^

The benefits (prevention of COVID-19 disease and associated hospitalizations, ICU admissions, and deaths) outweighed the risks (expected myocarditis cases after vaccination) in all populations for which vaccination has been recommended. However, the balance of benefits and risks varied by age and sex because cases of myocarditis were primarily identified among males aged <30 years, and the risks of poor outcomes related to COVID-19 increase with age. Per million second doses of mRNA COVID-19 vaccine administered to males aged 12–29 years, 11,000 COVID-19 cases, 560 hospitalizations, 138 ICU admissions, and six deaths due to COVID-19 could be prevented, compared with 39–47 expected myocarditis cases after COVID-19 vaccination (Table 2). Among males aged ≥30 years, 15,300 COVID-19 cases, 4,598 hospitalizations, 1,242 ICU admissions, and 700 deaths could be prevented, compared with three to four expected myocarditis cases after COVID-19 vaccination. This analysis did not include the potential benefit of preventing post-COVID-19 conditions, such as prolonged symptoms and MIS-C (*6*,*7*).

**TABLE 2:** Individual-level estimated number of COVID-19 cases and COVID-19–associated hospitalizations, intensive care unit admissions, and deaths prevented after use of 2-dose mRNA COVID-19 vaccine for 120 days and number of myocarditis cases expected per million second mRNA vaccine doses administered, by sex and age group* — United States, 2021

Sex/Benefits and harms from mRNA vaccination	No. per million vaccine doses administered in each age group (yrs)^†^				
	12–29	12–17	18–24	25–29	≥30
**Male**					
**Benefit**					
COVID-19 cases prevented^§^	11,000	5,700	12,100	15,200	15,300
Hospitalizations prevented	560	215	530	936	4,598
ICU admissions prevented	138	71	127	215	1,242
Deaths prevented	6	2	3	13	700
**Harms**					
Myocarditis cases expected^¶^	39–47	56–69	45–56	15–18	3–4
**Female**					
**Benefit**					
COVID-19 cases prevented^§^	12,500	8,500	14,300	14,700	14,900
Hospitalizations prevented	922	183	1,127	1,459	3,484
ICU admissions prevented	73	38	93	87	707
Deaths prevented	6	1	13	4	347
**Harm**					
Myocarditis cases expected^¶^	4–5	8–10	4–5	2	1

**Abbreviations:** ICU = intensive care unit; VAERS = Vaccine Adverse Event Reporting System.

* This analysis evaluated direct benefits and harms, per million second doses of mRNA COVID-19 vaccine given in each age group, over 120 days. The numbers of events per million persons aged 12–29 years are the averages of numbers per million persons aged 12–17 years, 18–24 years, and 25–29 years.

^†^ Receipt of 2 doses of mRNA COVID-19 vaccine, compared with no vaccination.

^§^ Case numbers have been rounded to the nearest hundred.

^¶^ Ranges calculated as ±10% of crude VAERS reporting rates. Estimates include cases of myocarditis, pericarditis, and myopericarditis.

ACIP also reviewed population-level considerations regarding vaccination. No alternatives to mRNA COVID-19 vaccines for adolescents will be available for the foreseeable future, and vaccination of adolescents offers protection against COVID-19 that can be important for returning to educational, social, and extracurricular activities. Higher levels of vaccination coverage can reduce community transmission, which can protect against development and circulation of emerging variants. Regarding health equity considerations, racial and ethnic minority groups have higher rates of COVID-19 and severe disease*****; potential changes in vaccine policy, or anything that would affect vaccination coverage for adolescents or young adults, might disproportionately affect those groups with the highest rates of poor COVID-19 outcomes.

The ACIP discussion concluded that 1) the benefits of vaccinating all recommended age groups with mRNA COVID-19 vaccine clearly outweigh the risks of vaccination, including the risk of myocarditis after vaccination; 2) continuing to monitor outcomes of myocarditis cases after COVID-19 vaccination is important; and 3) providers and the public should be informed about these myocarditis cases and the use of COVID-19 vaccines. Based on ACIP’s conclusion regarding the benefit-risk assessment on June 23, 2021, COVID-19 vaccination continues to be recommended for all persons aged ≥12 years under the FDA’s EUA. ACIP emphasized the importance of informing vaccination providers and the public about the benefits and the risks, including the risk for myocarditis after COVID-19 vaccination, particularly for males aged 12–29 years.

CDC has provided guidance regarding evaluation and management of myocarditis after mRNA COVID-19 vaccine (https://www.cdc.gov/vaccines/covid-19/clinical-considerations/myocarditis.html), as well as considerations for a second vaccine dose in persons who develop myocarditis after a first dose (https://www.cdc.gov/vaccines/covid-19/info-by-product/clinical-considerations.html). FDA has added information to the Pfizer-BioNTech^†††††^and Moderna^§§§§§^ COVID-19 vaccine EUA and fact sheets regarding myocarditis cases that have been reported among vaccine recipients. In addition, CDC has updated patient education and communication materials reflecting this information for the Pfizer-BioNTech^¶¶¶¶¶^ and Moderna****** COVID-19 vaccines; these are important to ensure that vaccine recipients, especially males aged 12–29 years, are aware of increased risk for myocarditis and to seek care if they develop symptoms of myocarditis. The vaccine product-specific EUA fact sheet should be provided to all vaccine recipients and their caregivers before vaccination with any authorized COVID-19 vaccine.

CDC and FDA will continue to closely monitor reports of myocarditis after receipt of the mRNA COVID-19 vaccines and will bring any additional data to ACIP for consideration. The benefit-risk analysis can be updated as needed to reflect changes in the COVID-19 pandemic and additional information on the risk for and outcomes of myocarditis after COVID-19 vaccination. The ACIP recommendation for use of mRNA COVID-19 vaccines under an EUA is interim and will be updated as additional information becomes available.

## Reporting of Vaccine Adverse Events

FDA requires that vaccine providers report to VAERS vaccination administration errors, serious adverse events,^††††††^ cases of multisystem inflammatory syndrome, and cases of COVID-19 that result in hospitalization or death after administration of a COVID-19 vaccine under an EUA. CDC also encourages reporting of any additional clinically significant adverse event, even if it is not clear whether a vaccination caused the event. Information on how to submit a report to VAERS is available at https://vaers.hhs.gov/index.html or 1-800-822-7967. In addition, CDC has developed a voluntary smartphone-based online tool (v-safe) that uses text messaging and online surveys to provide near real-time health check-ins after receipt of a COVID-19 vaccine. In cases of v-safe reports that include possible medically attended health events, CDC’s v-safe call center follows up with the vaccine recipient to collect additional information for completion of a VAERS report. Information on v-safe is available at https://www.cdc.gov/vsafe.

**Summary**

**What is already known about this topic?**

An elevated risk for myocarditis among mRNA COVID-19 vaccinees has been observed, particularly in males aged 12–29 years.

**What is added by this report?**

On June 23, 2021, the Advisory Committee on Immunization Practices concluded that the benefits of COVID-19 vaccination to individual persons and at the population level clearly outweighed the risks of myocarditis after vaccination.

**What are the implications for public health practice?**

Continued use of mRNA COVID-19 vaccines in all recommended age groups will prevent morbidity and mortality from COVID-19 that far exceed the number of cases of myocarditis expected. Information regarding the risk for myocarditis with mRNA COVID-19 vaccines should be disseminated to providers to share with vaccine recipients.

## Footnotes

* These authors contributed equally to this work.

† All EUA documents for COVID-19 vaccines, including fact sheets, are available at https://www.fda.gov/emergency-preparedness-and-response/coronavirus-disease-2019-covid-19/covid-19-vaccines.

§ ACIP recommendations for all COVID-19 vaccines are available at https://www.cdc.gov/vaccines/hcp/acip-recs/vacc-specific/covid-19.html.

¶ COVID-19 Vaccine Safety Technical Work Group Reports are available at https://www.cdc.gov/vaccines/acip/work-groups-vast/index.html.

**
https://www.cdc.gov/mis/hcp/index.html

††
https://www.ahajournals.org/doi/10.1161/CIR.0000000000000239?url_ver = Z39.88-2003&rfr_id = ori:rid:crossref.org&rfr_dat = cr_pub%20%200pubmed#d3e785

§§ Age was not reported for 32 patients, and sex was not reported for 14 patients.

¶¶
https://www.cdc.gov/vaccinesafety/ensuringsafety/monitoring/cisa/index.html

*** Data collection for race/ethnicity of myocarditis cases is ongoing.

†††
https://www.cdc.gov/vaccines/covid-19/info-by-product/janssen/risk-benefit-analysis.html

§§§ https://covid.cdc.gov/covid-data-tracker/#demographicsovertime. Data were used for the most recent week not subject to reporting delays prior to the ACIP meeting.

¶¶¶ https://gis.cdc.gov/grasp/COVIDNet/COVID19_3.html. Data were used for the most recent week not subject to reporting delays prior to the ACIP meeting.

****
https://gis.cdc.gov/grasp/COVIDNet/COVID19_5.html

†††† Because of uncertainty in the accuracy of myocarditis reporting, given that reviews are ongoing, and some cases might not have been reported yet, myocarditis reporting rates are presented as a range of values, calculated as ±10% of the observed reporting rates.

§§§§
https://www.cdc.gov/coronavirus/2019-ncov/science/science-briefs/fully-vaccinated-people.html

¶¶¶¶
https://covid.cdc.gov/covid-data-tracker/#variant-proportions

*****
https://covid.cdc.gov/covid-data-tracker/#demographics

†††††
https://www.fda.gov/media/144413/download

§§§§§
https://www.fda.gov/media/144637/download

¶¶¶¶¶
https://www.cdc.gov/vaccines/covid-19/info-by-product/pfizer/index.html

******
https://www.cdc.gov/vaccines/covid-19/info-by-product/moderna/index.html

††††††
https://vaers.hhs.gov/faq.html
